# Avian and human influenza virus compatible sialic acid receptors in little brown bats

**DOI:** 10.1038/s41598-017-00793-6

**Published:** 2017-04-06

**Authors:** Shubhada K. Chothe, Gitanjali Bhushan, Ruth H. Nissly, Yin-Ting Yeh, Justin Brown, Gregory Turner, Jenny Fisher, Brent J. Sewall, DeeAnn M. Reeder, Mauricio Terrones, Bhushan M. Jayarao, Suresh V. Kuchipudi

**Affiliations:** 1 Animal Diagnostic Laboratory, Department of Veterinary and Biomedical Sciences, The Pennsylvania State University, University Park, PA, USA; 2 Department of Physics, The Pennsylvania State University, University Park, PA USA; 3 Pennsylvania Game Commission, 2001 Elmerton Ave, Harrisburg, PA USA; 4 Department of Biology, Temple University, Philadelphia, PA USA; 5 Department of Biology, Bucknell University, Lewisburg, PA USA

## Abstract

Influenza A viruses (IAVs) continue to threaten animal and human health globally. Bats are asymptomatic reservoirs for many zoonotic viruses. Recent reports of two novel IAVs in fruit bats and serological evidence of avian influenza virus (AIV) H9 infection in frugivorous bats raise questions about the role of bats in IAV epidemiology. IAVs bind to sialic acid (SA) receptors on host cells, and it is widely believed that hosts expressing both SA α2,3-Gal and SA α2,6-Gal receptors could facilitate genetic reassortment of avian and human IAVs. We found abundant co-expression of both avian (SA α2,3-Gal) and human (SA α2,6-Gal) type SA receptors in little brown bats (LBBs) that were compatible with avian and human IAV binding. This first ever study of IAV receptors in a bat species suggest that LBBs, a widely-distributed bat species in North America, could potentially be co-infected with avian and human IAVs, facilitating the emergence of zoonotic strains.

## Introduction

With a wide host range, ability to undergo genetic recombination and cross species barrier, influenza A viruses (IAVs) continue to spread globally, causing huge economic losses to the poultry industry and threatening public health. Unlike the low pathogenic avian influenza viruses (LPAIVs), highly pathogenic avian influenza viruses (HPAIVs) cause very severe disease in gallinaceous poultry often leading to 100% mortality within 2–3 days^1^. HPAIVs of H5N1 subtype are of particular concern as certain contemporary Eurasian lineage H5N1 viruses can carry an alarming case fatality rate of up to 50% in humans^2^. Negative strand segmented RNA genomes contribute to the genetic variability of IAVs. In addition, IAVs can infect a wide range of avian and mammalian host species resulting in the emergence of novel subtypes with altered species tropism and/or virulence.

It is widely known that aquatic birds such as ducks, gulls, and shorebirds serve as a natural reservoir of most known IAVs^3^. IAVs are known to infect a wide range of avian and mammalian hosts, and it is highly likely that their host range could be broader than currently known, with more reservoirs to be revealed. For example, Northwest Atlantic gray seals have recently been suggested to be an endemically infected wild reservoir population for diverse IAVs^4^. From these natural reservoirs, IAVs can evolve into novel variants which can potentially lead to human pandemics. Influenza pandemics occur time to time with the most recent pandemic being in 2009. It is undisputed that the next influenza pandemic will happen, but the only question is when it will happen. Despite several reports investigating the basis of genetic variability of IAVs, the precise mechanism of pandemic IAV generation still remains an unresolved mystery. It is possible that additional IAV reservoirs are yet to be identified which will help to see the full picture of IAV ecology and evolution.

Bats (order: Chiroptera) are one of the ancient mammals, and their speciation occurred long before the development of most modern mammals. Bats are globally distributed, relatively long lived^5^ and represent approximately 24% of all known mammalian species. Further, bats are one of the most diverse families of mammals found in nearly every habitat/continent around the world except Antarctica. More importantly, certain Old World bat species are known to be natural reservoirs of zoonotic viruses that cause some of the deadliest diseases in humans including filoviruses (such as Ebola and Marburg viruses), lyssaviruses, severe acute respiratory syndrome (SARS)-related coronaviruses and henipaviruses (e.g. Hendra and Nipah viruses)^6, 7^. In addition, bats also act as a major natural reservoir for hepaciviruses and pegiviruses (hepatitis C virus and GB virus B)^8^. Notably, all the zoonotic viruses of bat-origin identified to date are RNA viruses^5^. However, it is believed that the actual diversity of viruses in bats could be much more than what is currently known, ﻿as most of the﻿ investigations have focused on searching for specific viruses of interest and many additional viruses must have been overlooked^9^.

The prospects for bats contributing to IAV epidemiology came to light after the identification of two novel influenza-like viruses in fruit bats by next generation sequencing^10^. These two viruses are genetically distinct from all previously known IAVs and hence are designated as novel subtypes, namely H17N10 and H18N11. These novel IAVs have been recently recovered in cell culture using synthetic DNA^11^. However, these HA and NA subtypes have not been identified in birds serologically or virologically. Consequently, the reservoir(s) of these novel IAV subtypes is still undefined. Phylogenetic studies raised a possibility that bats have the capacity to harbor more influenza virus genetic diversity than all the other mammalian and avian species combined^10^. In addition, it was demonstrated that little yellow-shouldered bats in Central America could constitute a potential sylvatic mammalian reservoir of influenza^12^. Susceptibility of bats to IAVs has been confirmed by recent serological evidence of AIV H9 subtype in about 30% of frugivorous bats from Africa^13^. It is worth noting that detection of antibodies against one AIV subtype in 30% of the bats tested is very significant.

Influenza viruses bind to sialic acid (SA) residues that are bound to glycans through α2,3 or α2,6 linkage on the host cells^14^. The expression of the appropriate host cell receptor to which viral haemagglutinin (HA) can bind is the key determinant of the ability of IAVs to infect a host^15^. Avian influenza viruses (AIVs) preferentially bind to SA receptors that are linked to galactose by an α 2,3 linkage (SA α2,3-Gal), while human and classical swine influenza viruses show preference to α2,6 linked SAs (SA α2,6-Gal). A key source of IAV genetic diversity could be from the replication of IAVs in a non-native host species that initiate evolution of new virus variants^16^. Hosts that co-express both SA α2,3-Gal and SA α2,6-Gal receptors such as chickens, ducks and pigs have been hypothesized to potentially support re-assortment of IAVs and hence play a major role in the evolution of IAVs^14, 17^.

While the role of bats in IAV evolution is not yet known, recent evidence raises a primary question, “can bats support co-infection of avian and human IAVs?”. Consequently a logical first unknown which needs to be addressed is whether bats express appropriate SA receptors compatible with avian and human IAV binding. To resolve this enigma, we investigated for the first time the distribution of SA receptors in little brown bats (LBBs) (*Myotis lucifugus*), a widely-distributed bat species in North America and their compatibility to support avian and human IAV binding.

## Results

Tissues sections from a total of 10 juvenile and 10 adult LBBs were subjected to lectin histochemistry for the detection of influenza virus receptors. No differences in SA receptor distribution between the juvenile and adult LBBs were found.

### Co-expression of both SA α2,3-Gal and SA α2,6-Gal receptors in LBB respiratory tract

**Figure 1:**
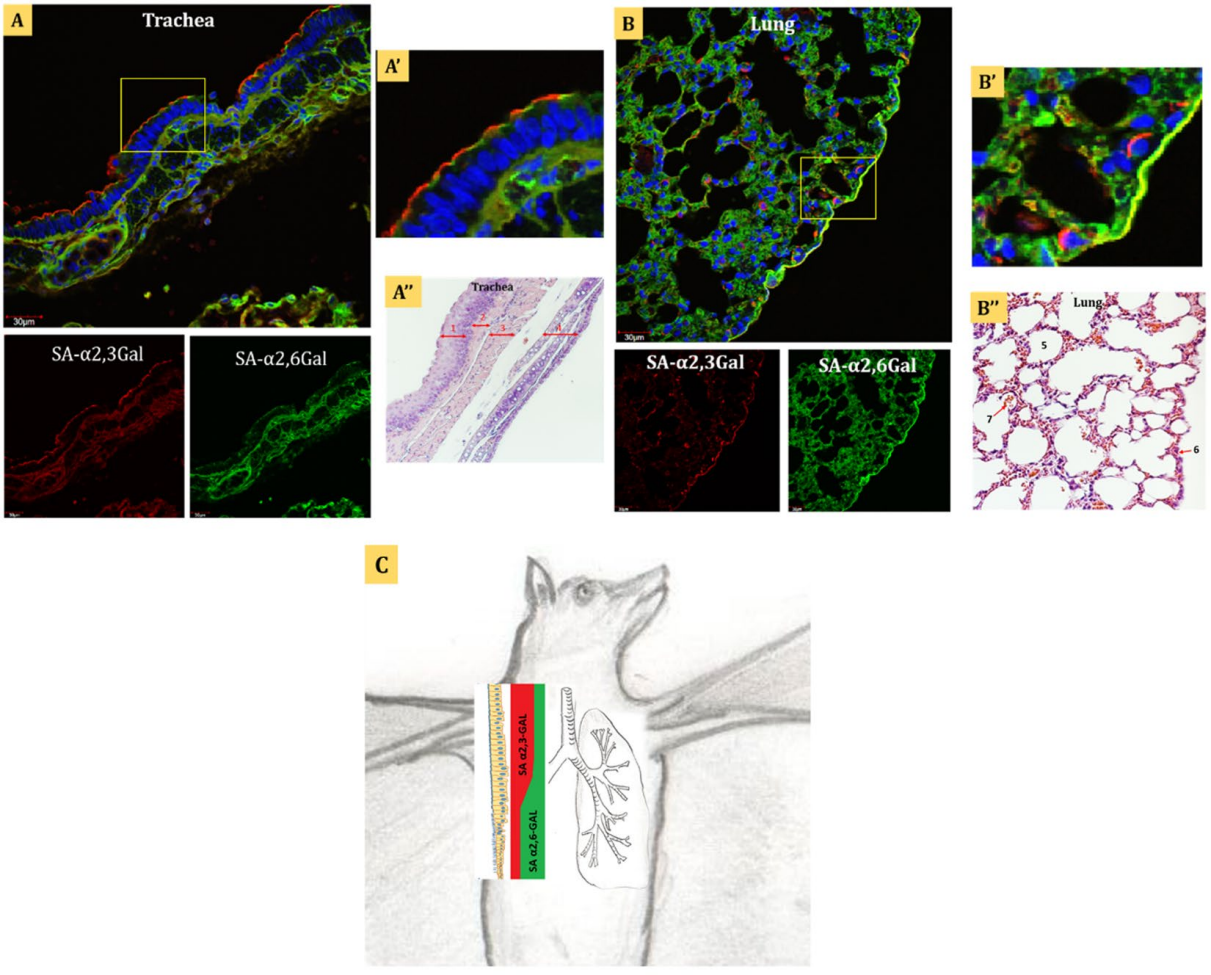
Co-expression of SA α2,3-Gal and SA α2,6-Gal receptors in little brown bat respiratory tract. Composite confocal images show co-expression of both SA α2,3-Gal (red) and SA α2,6-Gal (green) influenza receptors on the (**A**) trachea and (**B**) lung of the little brown bat (LBB). The SA α2,3-Gal receptor is the predominant receptor type on the (A’) respiratory mucosal epithelium of the trachea, while the SA α2,6-Gal receptor type is predominant in the lamina propria and submucosa of the trachea and lungs. (**C**) Schematic representation of the SA receptor distribution in the respiratory system of LBB shows predominance of SA α2,3-Gal receptor type in the upper respiratory tract, which gradually decreases towards the lower respiratory tract. In contrast, SA α2,6-Gal receptors gradually increases towards the lower respiratory tract. Tissue sections were stained with biotinylated MAAII (red - specific for avian type receptor, SA α2,3-Gal) and FITC labelled SNA (green - specific for human type receptor, SA α2,6-Gal) lectins, and DAPI nuclear stain (blue). A”: haematoxylin and eosin (H and E) stained tracheal tissue section. 1. respiratory epithelium, 2. lamina propria, 3. submucosa, 4. hyaline cartilage B”: H and E stained lung tissue section. 5. alveolar duct, 6. visceral pleura, 7. pulmonary blood capillary.

Abundant co-expression of avian (SA α2,3-Gal) and human (SA α2,6-Gal) type influenza receptors was found throughout the LBB respiratory tract (Fig. 1).

The relative abundance of avian and human type influenza receptors were distinctly different in the upper and lower airway of LBB. While SA α2,3-Gal receptors were predominant on the mucosal lining of the trachea (Fig. 1A), predominance of SA α2,6-Gal receptors was found in the alveolar epithelium, alveolar duct and visceral pleura of the lung (Fig. 1B). Further, abundant expression of SA α2,6-Gal receptors was found in the lamina propria and submucosa of the tracheal tissue (Fig. 1A).

### Predominant expression of SA α2,3-Gal receptors throughout LBB digestive tract

**Figure 2:**
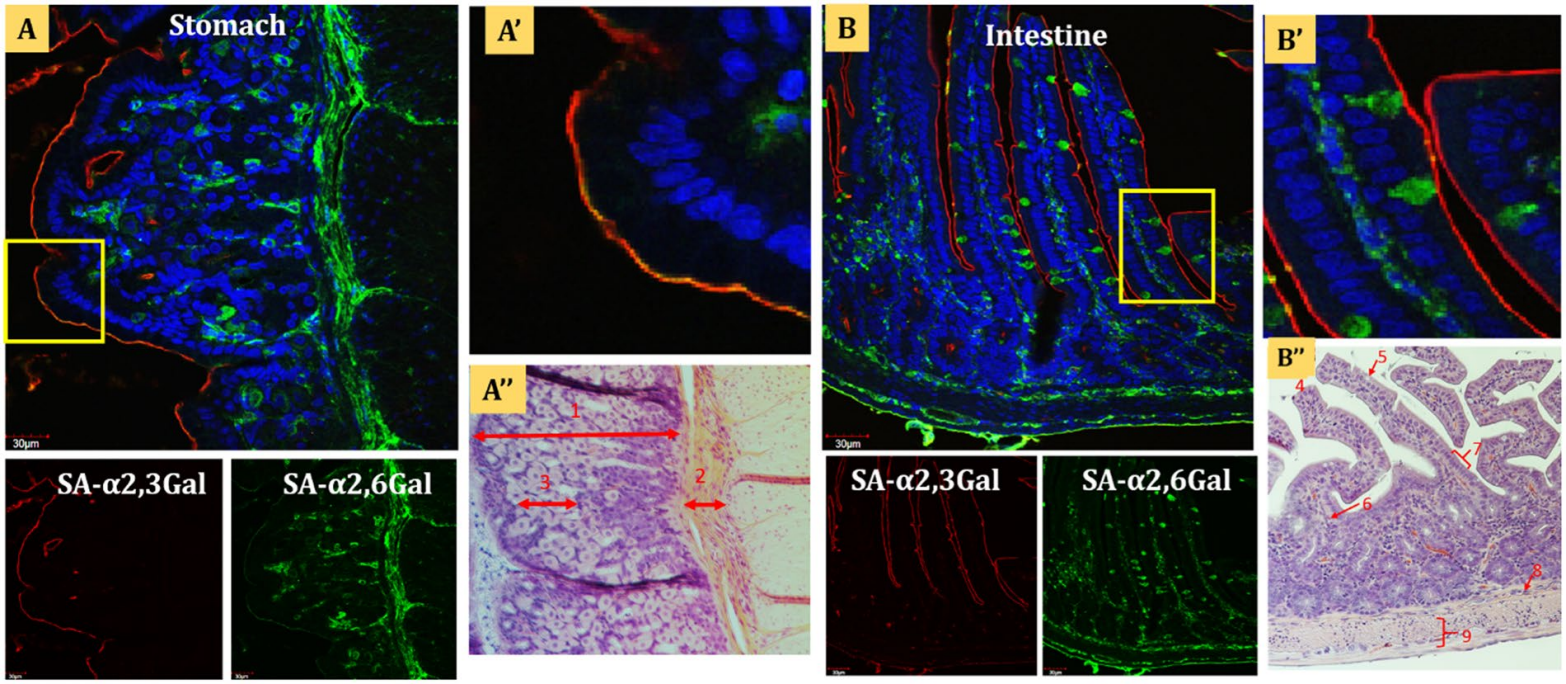
Co-expression of SA α2,3-Gal and SAα2,6-Gal receptors in LBB digestive tract. Composite confocal images show co-expression of both SA α2,3-Gal (red) and SA α2,6-Gal (green) influenza receptors on the mucosal lining of (**A**) stomach and (**B**) intestine of the little brown bat (LBB). The SA α2,3-Gal receptor is the predominant receptor type on the mucosal lining of the (A’) stomach and (B’), intestinal villi SA α2,6-Gal receptor type is more prominent in the lamina propria, muscularis and serosa of the (**A**) stomach and (**B**) intestine. Tissue sections were stained with biotinylated MAAII (red - specific for avian type receptor, SA α2,3-Gal) and FITC labelled SNA (green - specific for human type receptor, SAα2,6-Gal) lectins, and DAPI nuclear stain (blue). A”: h aematoxylin and eosin (H and E) stained stomach tissue section. 1. mucosa 2. submucosa 3. mucosal glands. B”: H and E stained intestinal tissue section 4. villus, 5. goblet cell, 6. lamina propria, 7. simple columnar epithelial cell layer, 8. submucosa 9. muscularis.

Predominant expression of SA α2,3-Gal receptors was found in the stomach and intestines of LBB (Fig. 2). Cells lining the mucosa of stomach exhibited co-expression of both avian and human receptors (Fig. 2A). The luminal epithelium of intestines primarily expressed SA α2,3-Gal receptors whereas considerable expression of SA α2,6-Gal receptors were found on the goblet cells, lamina propria, muscularis and serosa of the intestine (Fig. 2B).

### SA α2,3-Gal receptors in LLBs are compatible with avian H5N2 virus binding

**Figure 3:**
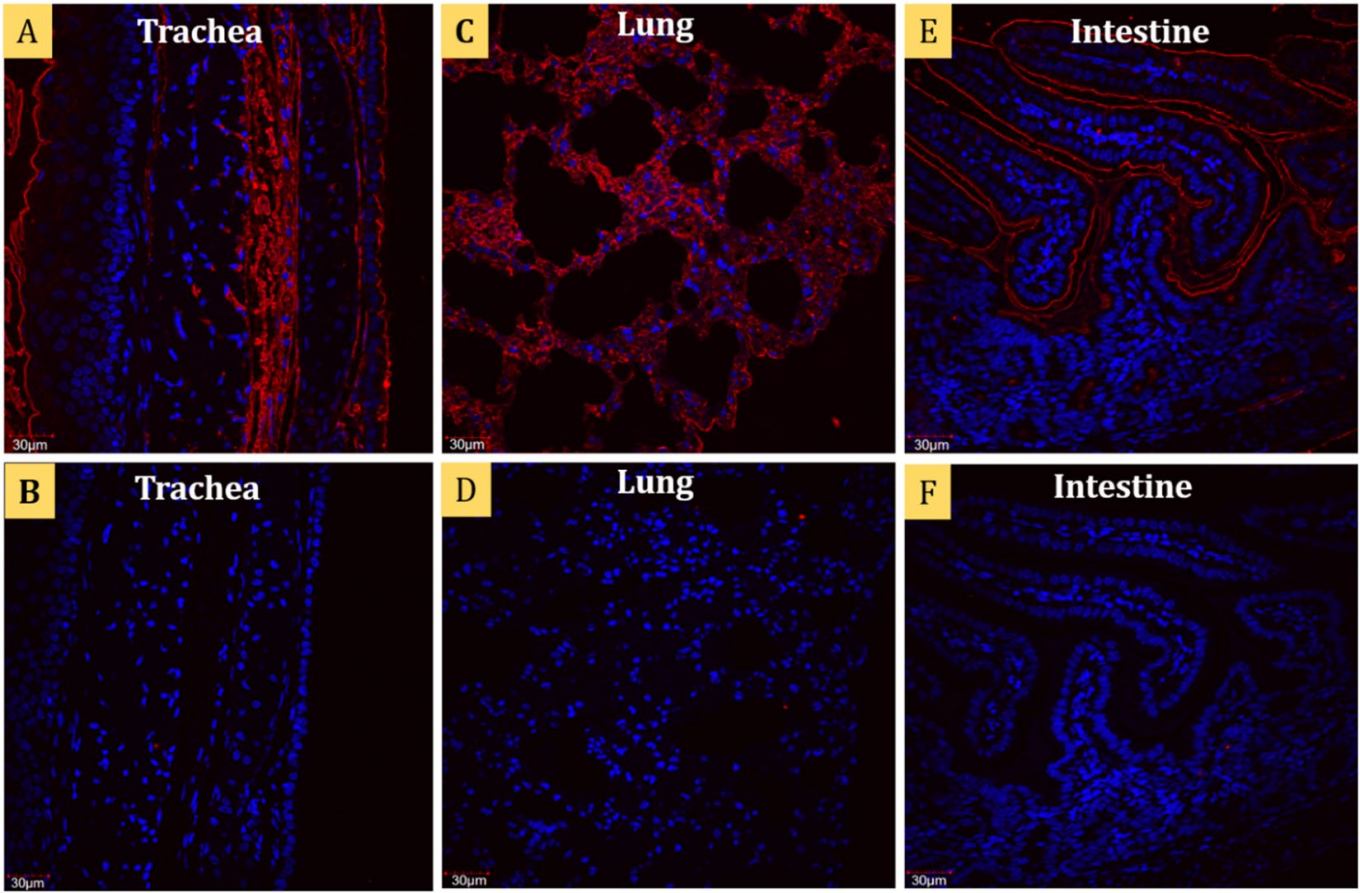
Widespread binding of avian H5N2 virus to LBB tissues. The confocal images show extensive binding of avian H5N2 virus (A/H5N2/chicken/Pennsylvania/7659/1985) to little brown bat (LBB) (**A**) trachea, (**C**), lung and (**E**) intestine. The virus binding pattern reflects the relative abundance of the SA α2,3-Gal receptors in the trachea, lung and intestine of the little brown bat. The respiratory epithelial cells of trachea and luminal mucosal cells of the intestine show preferential binding to the influenza virus. The lung demonstrates partial virus binding as shown by Cy-5 labeled secondary antibody. (**B**, **D** and **F**) Mock treated trachea, lung and intestine respectively.

Virus binding assays were performed on LBB trachea and lung sections using an LPAIV H5N2 (A/H5N2/chicken/Pennsylvania/7659/1985) virus. Extensive binding of avian H5N2 virus to LBB trachea, lung and intestines was observed (Fig. 3). H5N2 virus binding pattern was in accordance with the relative abundance of SA α2,3-Gal receptors in tissues such that greater virus binding to the tracheal (Fig. 3A) and intestinal mucosa (Fig. 3E) was observed compared to the lung (Fig. 3C). Mock-treated tissues (Fig. 3B, D and F) did not exhibit any background staining.

### SA α2,6-Gal receptors in LLBs are compatible with human H1N1 virus binding

**Figure 4:**
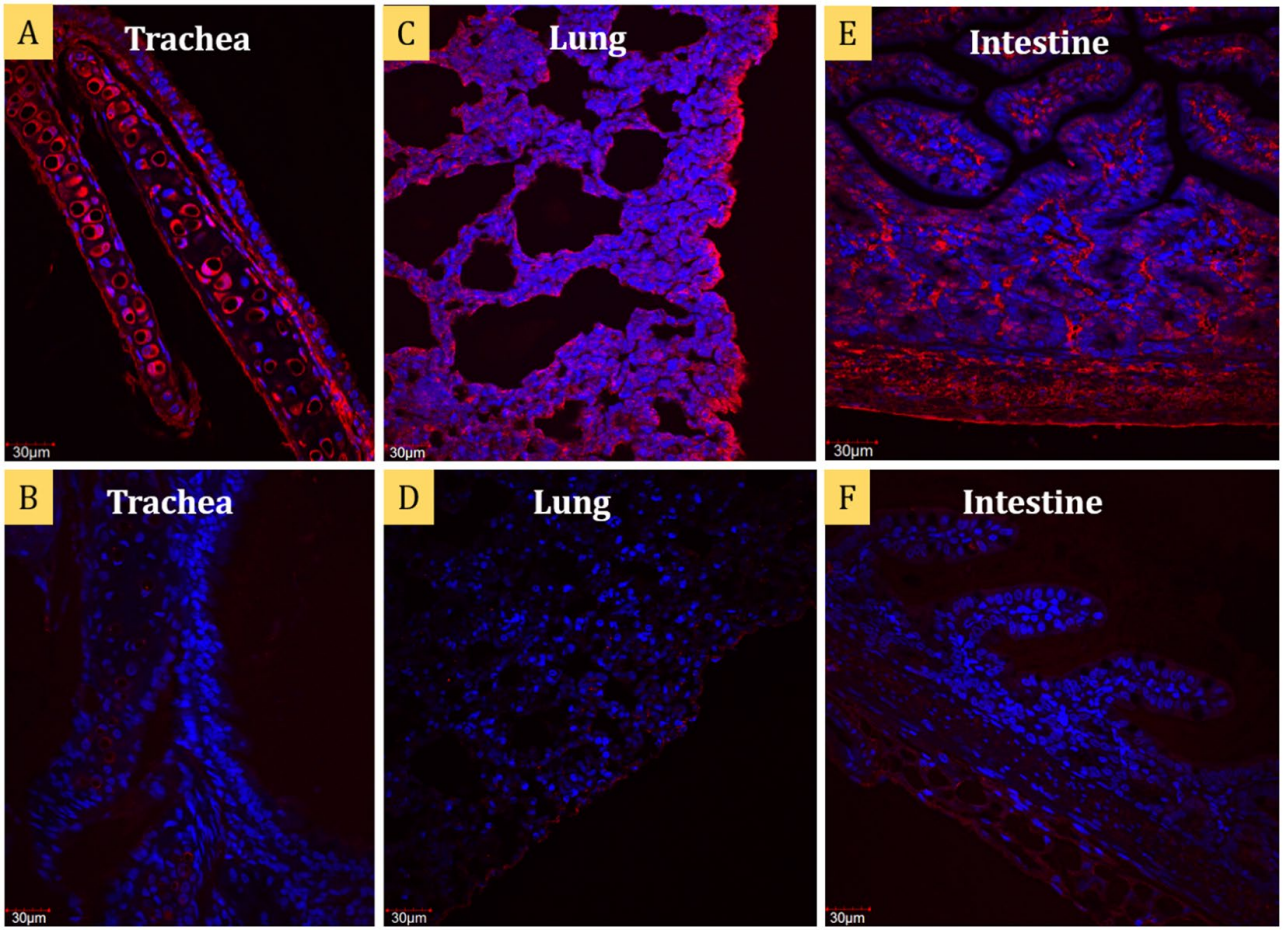
Widespread binding of human pandemic H1N1 virus to LBB tissues. Confocal images show extensive binding of human H1N1 virus (A/H1N1/Virginia/2009) to little brown bat (LBB) (**A**) trachea, (**C**), lung and (**E**) intestine. Binding of human pandemic H1N1 virus was in accordance with the abundance of SA α2,6-Gal receptors in these tissues. Virus binding was observed predominantly in the submucosa and the hyaline cartilage of the (**A**) trachea, alveolar epithelial cells of the (**C**) lung and to lamina propria, muscularis and serosa of the (**E**) intestine. (**B**, **D** and **F**) mock treated trachea, lung and intestine respectively.

Extensive binding of human H1N1 (A/H1N1/Virginia/2009) virus to LBB trachea, lung and intestine was observed (Fig. 4). As expected, greater binding of H1N1 virus was observed in the lungs (Fig. 4C) which had greater SA α2,6-Gal expression. Notably, very little virus binding was found on the mucosal lining of intestinal villi (Fig. 4E) which had primarily SA α2,3-Gal receptor expression. However, a fair amount of virus binding to the intestinal submucosa was observed. Widespread binding of the H1N1 virus to tracheal mucosa was observed (Fig. 4A). Mock tissues (Fig. 4B, D and F) did not exhibit any background staining.

**Figure 5:**
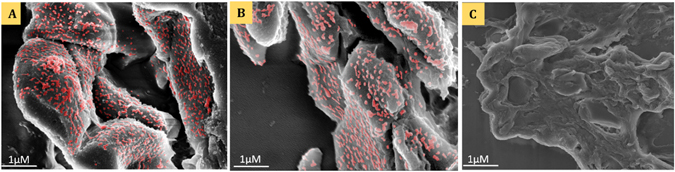
Scanning electron micrograph showing virus binding. Scanning electron micrograph showing binding of (**A**) avian H5N2 virus (A/H5N2/chicken/Pennsylvania/7659/1985) and (**B**) human H1N1 virus (A/H1N1/Virginia/2009) to little brown bat (LBB) trachea. (**C**) Mock controls were performed omitting virus incubation. Extensive binding of avian H5N2 and human H1N1 viruses to LBB trachea was observed. Virus or mock treated sections were fixed in ethanol and were coated with gold before imaging by a field emission SEM (FE-SEM, Merlin Zeiss) under 5 eV. Virus particles were pseudo-colored using Adobe Photoshop CC.

In addition to the virus binding assay using antibody-based detection, we also visualized virus binding using scanning electron microscopy (SEM) which also confirmed abundant binding of avian H5N2 virus (Fig. 5A) and human H1N1 virus (Fig. 5B) to LBB trachea (Fig. 5).

### Sialidase A treatment abrogated lectin and virus binding

It has been shown that lectins from different suppliers may show different binding specificities; in particular the source of MAA has been shown to significantly affect specificity. Lectins (SNA and MAAII) used in this study were purchased from Vector Laboratories which were shown to bind to the appropriate sialic acid linkages with a high degree of specificity by Glycan microarray screening (http://www.functionalglycomics.org). We treated sections with Sialidase A (N-acetylneuraminate glycohydrolase; Prozyme, San Leandro, CA), for 2 h at 37°C, which cleaves all non-reducing terminal sialic acid residues in the order α(2,6) > α(2,3) > α(2,8) > α(2,9). Sialidase A treatment completely abrogated lectin binding (Supplementary Figure S1) and H5N2 influenza virus binding (Supplementary Figure S2) which further confirmed the specificity of the lectins used in this study to appropriate sialic acid linkages.

In summary, we found that the SA receptors on LBB tissues supported binding of avian H5N2 and human H1N1 influenza viruses. Further, the virus binding pattern was in accordance with the relative distribution of SA α2,3-Gal and SA α2,6-Gal receptors in tissues.

## Discussion

With the continued recognition of the role of various wild animals in influenza virus evolution, the interest in understanding the tissue SA distribution in wild animals has intensified^18^. Among the various factors, the ability to bind to SA receptors on host cells is considered a key feature of IAV pathogenicity. There is serological evidence of avian influenza virus infection in certain frugivorous bats^13^ and *Pteropus alecto* bat cells were found to be susceptible to IAV infection and reassortment^19^. In addition bats have been shown to harbor novel influenza-like viruses^10^. However, to date, the SA receptor distribution in any bat species is completely unknown. For the first time, this study demonstrated that little brown bats (LBBs), a widely-distributed bat species in North America, co-express both avian and human type influenza receptors in their respiratory and gastrointestinal systems.

Co-expression of avian and human type influenza receptors was found in LBB trachea and lung. However avian type (SA α2,3-Gal) receptors were predominant in tracheal mucosa similar to ducks^14^ and some species of Passeriformes and Charadriiformes^20^. In contrast SA α2,6-Gal receptors are predominant in the tracheal mucosal lining in chickens, pigs and humans^14, 17^. We found co-expression of both avian and human type receptors in the mucosal lining of stomach and to a lesser degree in intestines. SA α2,3-Gal receptors were predominant throughout the digestive tract much like chickens and ducks^14, 21, 22^. In contrast, other mammalian species such as humans and pigs predominantly express SA α2,6-Gal receptors in the GI tract^17^. Unlike chickens and ducks, low levels of SA α2,6-Gal receptor expression was also observed in LBB digestive tract which decreased progressively from stomach to intestines. We did not find any difference in the receptor distribution among various locations in intestines as it is difficult to distinguish large and small intestine in most bat species^23^. Similarly, no major differences in the receptor distribution pattern between large and small intestine were found in most other species including chickens, ducks and pigs^14, 17^. A fair amount of SA α2,6-Gal receptor expression was found in the intestinal crypts, lamina propria, muscularis and serosa of LBB intestine. It is worth noting that a similar co-expression pattern of influenza receptors is observed in various wild birds of the families Columbiformes, Anseriformes and Gruiformes^20^, which constitute the natural influenza virus reservoir, and also in pigs^17^. Virus binding assays with an avian H5N2 and a human H1N1 virus confirmed that the SA receptors found in LBB tissues are compatible with avian and human IAV binding.

Bats act as natural reservoirs for a variety of zoonotic viruses and they coexist with viruses through several mechanisms including elevated metabolism and body temperature^24^. It is believed that this unique feature of bats leads to the selection of viruses that adapt better at higher body temperature, and hence are more virulent to humans^24^. Bats carry a number of RNA and DNA viruses asymptomatically, and the detection rate of new viruses or virus sequences seems to be much higher in bats than any other mammals^25^. The perfect equilibrium between various zoonotic viruses and bats have been studied extensively in the past two decades^7, 26^.

IAVs have been isolated from more than 105 different bird species belonging to 26 different families^27^. Although Anseriforms (ducks, geese, and swans) and Charadriiforms (gulls, terns, and shorebirds) are considered to be the major influenza reservoirs, IAVs have also been isolated from Gaviiformes (loons), Podicepediformes (grebes), Procellariiformes (shearwaters and petrels), Pelecaniformes (pelicans and cormorants), Ciconiiformes (ibis and herons), and Gruiformes (moorhen and coots)^28^. Many of these species may indeed be reservoirs of IAVs, but no systematic surveys have been conducted, leaving a gap in our current understanding of the natural reservoirs of IAVs.

We showed for the first time that LBBs co-express both the avian (SA α2,3-Gal) and human (SA α2,6-Gal) influenza receptors in their respiratory and gastrointestinal system that are compatible with avian H5N2 and human H1N1 virus binding. In addition, there is strong evidence that cell lines from a range of bat species support productive IAV replication^16^. The sum of this evidence suggests that bats could play an important role in IAV epidemiology and zoonotic IAV emergence.

As the novel bat influenza viruses are different from other IAVs, it was proposed that these viruses would therefore require significant changes before they can infect and spread among humans^11^. However, a recent study rescued these viruses using reverse genetics in cell culture and found that the novel bat influenza viruses can infect a range of mammalian cells including canine cells^29^. All the existing data suggests that bats could be susceptible to many different IAV subtypes and even support co-infection of avian and human IAVs. It is believed that the novel bat influenza viruses found in fruit bats are probably the ancient influenza viruses from which the modern world IAVs have been derived over time^30^. Evidence of high seroprevalence of avian influenza in frugivorous bats together with the evidence of abundant SA receptors in LBBs found in this study, raises a strong possibility that bats could be a major influenza virus reservoir.

Despite many rigorous scientific pursuits, we have been unable to understand the mechanism by which new pandemic influenza viruses emerge. Consequently, we do not yet have sufficient scientific understanding needed to accurately predict which IAV strains may cause the next pandemic. The extensive diversity of bat species globally and the limited understanding of the role of bats in IAV biology raises an urgent need for comprehensive epidemiological surveillance of IAVs across different bat species.

## Materials and Methods

### Experimental animals

Little brown bat respiratory and gastrointestinal tissues were collected and provided by PA Game Commission from an ongoing collaborative study with Bucknell University, on little brown bats from the state of Wisconsin and New York. The study (DMR-17) was approved by the Bucknell University Institutional Animal Care and Use Committee and all methods were carried out in accordance with relevant guidelines and regulations. A total of 10 adult and 10 juvenile male little brown bat tissues were included in the study.

### Lectin histochemistry

Lectin histochemistry on paraffin embedded tissues was performed as previously described^14^. Briefly, 5 µm thick tissue sections were deparaffinized in xylene and rehydrated in ethanol. Following 10 min presoaking of the rehydrated sections in Tris-buffered saline (TBS), sections were incubated with biotinylated Maackia amurensis (MAAII) and FITC labelled Sambucus Nigra (SNA) lectins specific to SA α2,3-Gal and SA α2,6-Gal receptor respectively each at a concentration of 10 μg/ml, overnight at 4°C. Both lectins were purchased from Vector laboratories (Burlingame, CA, USA). Following three washes with TBS, sections were incubated with Streptavidin Alexafluor 594 conjugate for 2 h at 4°C. The sections were washed three times with TBS and mounted in ProLongGold antifade reagent with nuclear stain 4′,6-diamino-2-phenylindole, dihydro-chloride (DAPI). Negative controls were performed omitting the primary reagents. Following 24 h of curing at room temperature (RT), sections were imaged using Olympus FluoView™ FV1000 confocal microscope. Settings of the confocal microscope were determined using negative controls to avoid any background fluorescence and the same settings were used to scan all the other sections for consistency. To rule out nonspecific binding of the lectins and IAVs, control tissue sections were treated, prior to lectin staining or virus binding, with Sialidase A (N-acetylneuraminate glycohydrolase; Prozyme, San Leandro, CA), for 2 h at 37°C, which cleaves all non-reducing terminal sialic acid residues in the order α(2,6) > α(2,3) > α(2,8) > α(2,9). Sialidase treated and control sections were further subjected to lectin staining or virus binding.

### Virus binding assay

Virus binding assays with a low pathogenic AIV (LPAIV) H5N2 (A/H5N2/chicken/Pennsylvania/7659/1985) and human pandemic H1N1 virus (A/H1N1/Virginia/2009) were performed as previously described^14^. Virus binding assays were performed following strict biosafety level-2 (BSL-2) practices. Briefly deparaffinised tissue sections were incubated with a 250 μl of LPAI H5N2 virus (10^6^ TCID_50_/ml) or human H1N1 virus (10^6^ TCID_50_/ml) for 2 h at RT. Tissues incubated with PBS served as mock treated controls. Sections were washed with TBS before blocking with inactivated goat serum for 30 min and immunostained with primary mouse monoclonal antibody to influenza hemagglutinin H5 (ab82455, Abcam) or influenza nucleoprotein (ab20343, Abcam). Following 40 min of incubation with the primary antibody, sections were washed and incubated with a secondary goat anti mouse IgG-Cy5 antibody (ab6563, Abcam). After 40 min of incubation with secondary antibody at RT, sections were washed with TBS and mounted in ProLongGold antifade reagent with DAPI. Following 24 h of curing, the sections were imaged using Olympus FluoView™ FV1000 confocal microscope.

### Scanning electron microscopy (SEM)

Virus binding was also visualized using scanning electron microscopy. Deparaffinized sections were incubated with LPAI H5N2 virus or human H1N1 virus for 2 h as described above. Subsequently the sections were washed with distilled water and fixed using increasing ethanol concentrations (50%, 70%, 90% and 100%). The sections were then dried by a critical point dryer (Leica EM CPD300). Gold was coated over the dried sections by applying sputter coating for 10 seconds (Cressington, 108 auto sputter coater) and SEM images were taken by a field emission SEM (FE-SEM, Merlin Zeiss) under 5 eV. Image processing and pseudo coloring of virus particles bound to tissues was performed using Adobe Photoshop CC.

## Electronic supplementary material

Supplemnetary figures
