# Supplementary material for: Avian and human influenza virus compatible sialic acid receptors in little brown bats
Source: Sci Rep. 2017 Apr 6;7:660. doi: 10.1038/s41598-017-00793-6 (PMC5429623; doi:10.1038/s41598-017-00793-6)
Supplement: Supplementary file 1 — Supplemnetary figures [file 41598_2017_793_MOESM1_ESM.pdf]

Supplementary figures

Avian and human influenza virus compatible sialic acid receptors in little brown bats

Shubhada K Chothe[1], Gitanjali Bhushan[1], Ruth H. Nissly[1], Yin-Ting Yeh[2], Justin Brown [1, 3], Gregory Turner[3], Jenny Fisher[1], Brent J. Sewall[4], DeeAnn M. Reeder[5], Mauricio Terrones[2], Bhushan M Jayarao[1] and Suresh V Kuchipudi[1*]

[1] Animal Diagnostic Laboratory, Department of Veterinary and Biomedical Sciences, The Pennsylvania State University, University Park, USA
[2] Department of Physics, The Pennsylvania State University, USA
[3] Pennsylvania Game Commission, 2001 Elmerton Ave, Harrisburg, PA, USA
[4] Department of Biology, Temple University, Philadelphia, PA, USA
[5]Department of Biology, Bucknell University, Lewisburg, PA, USA

[*]corresponding author email: skuchipudi@psu.edu

Figure S1

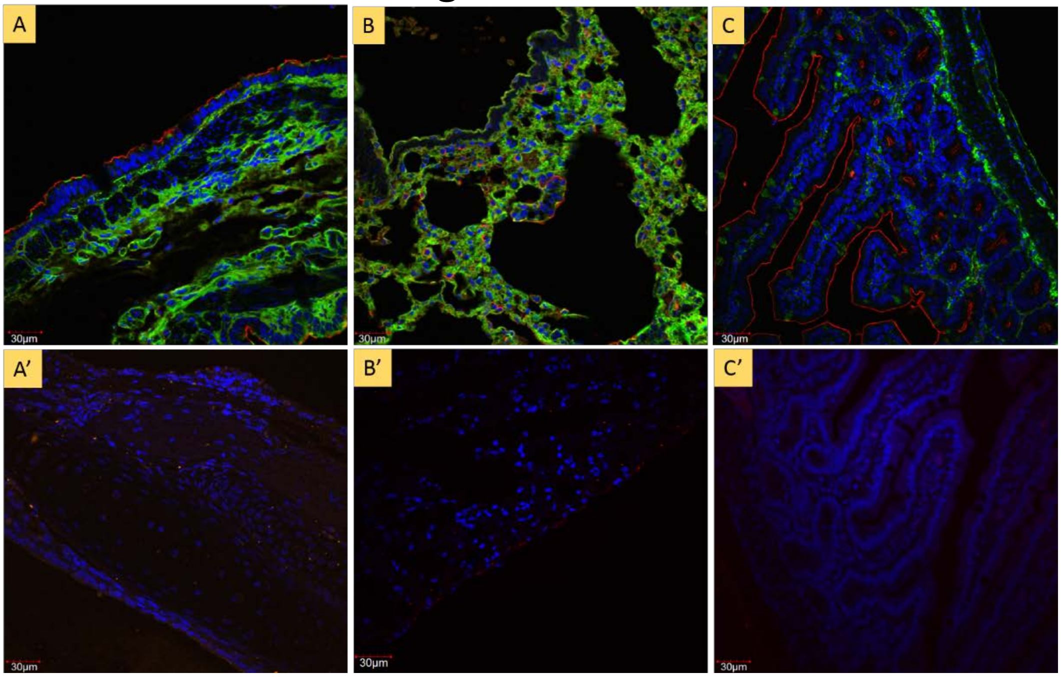

Figure S1: Sialidase A treatment of LBB tissue sections completely abrogated lectin binding. (A') Trachea (B') Lung and (C') intestine sections showing no lectin binding following Sialiadse A treatment. In contrast mock treated (A) Trachea (B) Lung and (C) intestine sections show abundant binding of SNA and MAAII lectins. Tissues sections were treated with Sialiadse A or buffer for 2hr at 37°C before lectin staining.

Figure S2

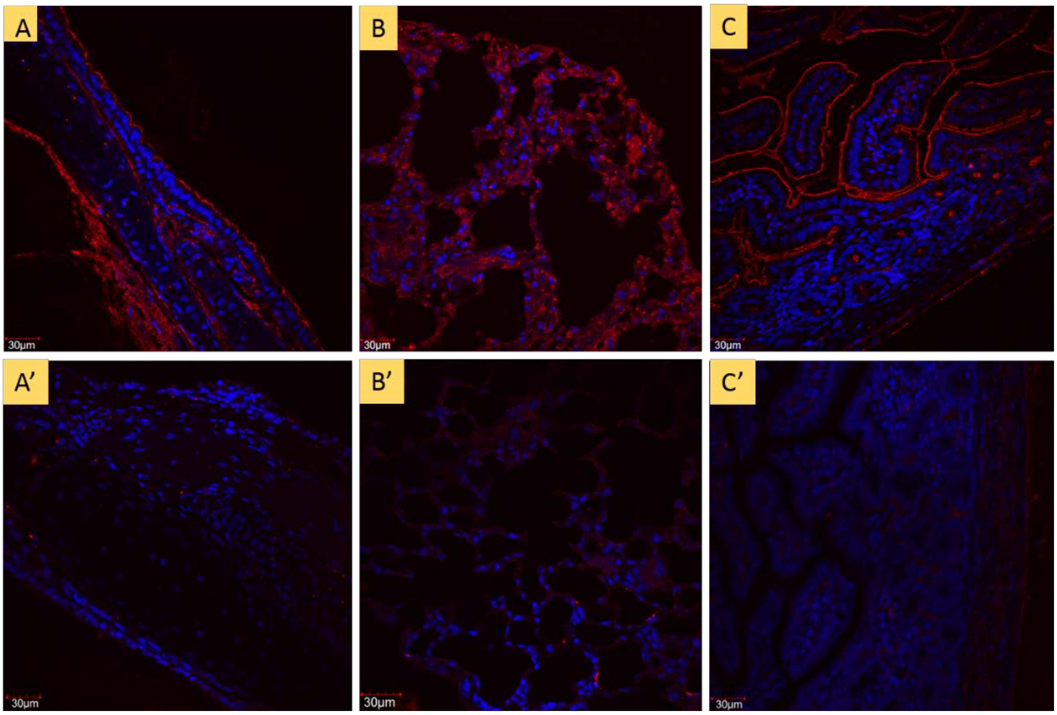

Figure S2: Sialidase A treatment of LBB tissue sections completely abrogated H5N2 influenza virus binding. (A') Trachea (B') Lung and (C') intestine sections showing no binding of LPAI H5N2 virus following Sialiadse A treatment. In contrast mock treated (A) Trachea (B) Lung and (C) intestine sections show abundant binding of H5N2 virus. Tissues sections were treated with Sialidase A or buffer for 2hr at 37°C before incubating with virus.
